# A self-photoprotection mechanism helps *Stipa baicalensis* adapt to future climate change

**DOI:** 10.1038/srep25839

**Published:** 2016-05-10

**Authors:** Xiliang Song, Guangsheng Zhou, Zhenzhu Xu, Xiaomin Lv, Yuhui Wang

**Affiliations:** 1 State Key Laboratory of Vegetation and Environmental Change, Institute of Botany, Chinese Academy of Sciences, 20 Nanxincun, Xiangshan, Beijing 100093, China; 2 Chinese Academy of Meteorological Sciences, China Meteorological Administration, 46 Zhongguancun South Street, Haidian, Beijing 100081, China; 3 University of Chinese Academy of Sciences, 19A Yuquan Road, Beijing 100049, China

## Abstract

We examined the photosynthetic responses of *Stipa baicalensis* to relative long-term exposure (42 days) to the predicted elevated temperature and water availability changes to determine the mechanisms through which the plant would acclimate to future climate change. Two thermal regimes (ambient and +4 °C) and three irrigation levels (partial, normal and excess) were used in environmental control chambers. The gas exchange parameters, light response curves and *A*/*C*_i_ curves were determined. The elevated temperature and partial irrigation reduced the net photosynthetic rate due to a limitation in the photosynthetic capacity instead of the intercellular CO_2_ concentration. Partial irrigation decreased Rubisco activation and limited RuBP regeneration. The reduction in *V*_cmax_ increased with increasing temperature. Excess irrigation offset the negative effect of drought and led to a partial recovery of the photosynthetic capacity. Although its light use efficiency was restricted, the use of light and dark respiration by *Stipa baicalensis* was unchanged. We concluded that nonstomatal limitation was the primary reason for photosynthesis regulation in *Stipa baicalensis* under relative long-term climate change conditions. Although climate change caused reductions in the light use efficiency and photosynthetic rate, a self-photoprotection mechanism in *Stipa baicalensis* resulted in its high ability to maintain normal live activities.

In a comparison with the 50 years from 1850 to 1900, the Fifth Assessment Report (AR5) of Intergovernmental Panel on Climate Change (IPCC) predicted that the global temperature will increase by approximately 1.5–4.0 °C because of the increase in the concentration of CO_2_ by the end of the 21^st^ century^1^. Simultaneously, extreme precipitation events in the mid latitude of the Northern Hemisphere will occur more frequently than in the past^1^. Both temperature and water are important abiotic factors, and the changes in these factors significantly influences plant physiological and biochemical processes, which then affect plant growth^2^. Among the processes in plants, photosynthesis is the most sensitive to environmental stress^3^,^4^, and the efficiency of photosynthesis plays an important role in plant growth and crop yield. Therefore, an understanding of the responses of plant photosynthesis to changes in temperature or water availability is necessary for people to address scientifically the problems of future climate change.

The stress from the environment directly and/or indirectly influences a series of physiological and biochemical processes that affect photosynthesis in plants, which include a change in pigment complexes, destruction of chloroplast structures, limited enzyme activities and disturbance to electron transport^5^,^6^,^7^. Among the environmental stress factors, a water deficit affects plant photosynthesis through stomatal and nonstomatal limitations^8^. The stomatal limitation leads to a decrease in the net photosynthesis (*P*_n_) because with a water deficit, the stoma is closed and the declined stomatal conductance results in a substomatal CO_2_ concentration (*C*_i_) that is insufficient for photosynthesis. The nonstomatal limitations, including photosynthetic phosphorylation, regeneration of ribulose-1,5-bisphosphate (RuBP), carboxylation efficiency (CE), light capture capability (PSII activity), activation of Rubisco and the synthesis of ATP, can also explain the photosynthetic down-regulation caused by a water deficit^9^,^10^,^11^,^12^. The theories on the influences of stomatal and nonstomatal limitation on *P*_n_ under water stress conditions have always been in contrast. The controversy may be because of species-specific effects in response to the length and intensity of the water stress on plants^13^,^14^. Generally, stomatal limitation is the primary reason for the reduction in photosynthetic efficiency in mild to moderate drought stress^15^, whereas nonstomatal limitations play a more important role in limiting the ability to assimilate carbon under more severe drought conditions^16^. Additionally, the decline in the photosynthetic rate is primarily caused by stomatal limitation in the early stages of water stress, whereas the reduction in photosynthesis with long-term water stress may be caused by nonstomatal limitations^17^.

One physiological process that is highly sensitive to high temperature stress is photosynthesis^4^ because the PSII in the light-dependent reactions and the Rubisco activase in the dark-dependent reactions are two thermally sensitive components of the photosynthetic apparatus^18^. Although many studies have been conducted to investigate the response of photosynthesis to high temperature stress, the physiological and biochemical mechanisms of the photoinhibition caused by the heat remain debatable^4^,^19^. The activation state of Rubisco is the key factor for the limitation on photosynthesis, and the Rubisco activase is highly susceptible to high temperatures^20^. Moreover, high temperature stress can cause a large decrease in both the Rubisco activation^21^ and the RuBP carboxylation rate^22^ by inhibiting the Rubisco activase^23^. However, some other researchers also found that the inhibition of photosynthesis under heat stress might result from other limitations, including those on RuBP regeneration, electron transport, and photosystem II (PSII) and I (PSI)^24^,^25^,^26^. Among the factors to limit photosynthesis, the PSII was the primary site of heat damage in the photosynthetic process^27^ and was the most thermally labile component of the electron transport chain^4^. When temperatures exceed 45 °C, damage to the PSII is dramatic^28^. However, for most plants, moderate heat stress reduces the photosynthetic rate not by damaging the PSII^28^ but by increasing the PSI cyclic electron flow^26^ and the proton conductance of the thylakoid membranes in dark-adapted leaves^29^.

In many studies, high field temperatures and periods of drought often occur simultaneously, particularly in semiarid or drought-stricken areas^30^. Clearly, the outcome caused by changes in one particular climate factor can be significantly affected by changes in other factors^31^,^32^. Although the effects of elevated temperature and water availability on photosynthesis with each as a single factor have been extensively studied, relatively little is known about how plants respond to the interaction of these factors. Thus, the responses of photosynthesis (including stomatal and nonstomatal limitations) to the simultaneous elevation in temperature and decrease in water availability must be determined^33^.

The grass *Stipa* Linn. is the primary dominant and constructive species in the grasslands of northern China. Affected by the spatial heterogeneity of heat and precipitation, the *Stipa* Linn. has a regular zonal distribution from the east to the west of China. The species *Stipa baicalensis* (S. *baicalensis*) is one of the dominant and constructive *Stipa* Linn. species in the meadow grasslands of China, which plays an important role in animal husbandry^34^. The determination of the photosynthetic characteristics of S. *baicalensis* in response to the changes in temperature and water availability can provide a theoretical basis for the reactions to climate change in the meadow steppe. The present study was performed using environmental control chambers to maintain the temperature and artificial irrigation to maintain the water availability for S. *baicalensis* at the required levels. Three hypotheses would be tested in the study: (1) The photosynthetic efficiency of S. *baicalensis*is is reduced by nonstomatal limitation under the conditions of relative long-term moderate water treatments. (2) The photosynthetic capacity of S. *baicalensis* under water deficit is further decreased by elevated temperature through reducing the activation of Rubisco and limiting the regeneration of RuBP. And (3) S. *baicalensis* is vulnerable to environmental stress and has difficulty in adapting to future climate change. The aims of this work were to investigate the relative effects of stomatal and nonstomatal limitations on photosynthesis to changes in the temperature and the availability of water and to determine the dominant factor in the regulation of photosynthesis in S. *baicalensis*. To achieve these goals, single-leaf gas exchange parameters, light response curves and *A*/*C*_i_ curves were determined to examine the responses of the physiological and biochemical mechanisms that affect the assimilation of carbon by S. *baicalensis* under conditions of elevated temperature and a change in water availability.

## Results

### Soil relative water content (SRWC) and leaf water potential (LWP)

**Figure 1:**
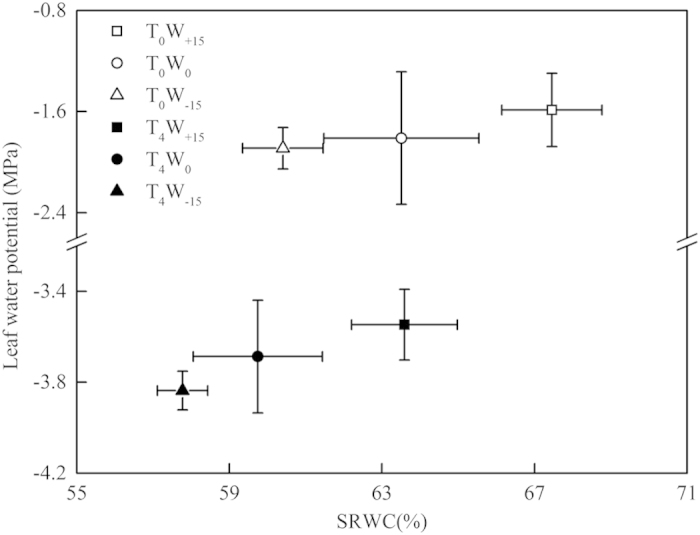
The change in soil relative water content (SRWC) and leaf water potential (LWP) in *Stipa baicalensis* under different temperature and water availability treatments.

The changes in the SRWC and the LWP of S. *baicalensis* in the different temperature and water availability treatments are shown in Fig. 1. At T_0_, the SRWC in the different treatments for water availability (W_+15_, W_0_ and W_−15_) was 67.5%, 63.5% and 60.4%, respectively. With the increase in temperature in the T_4_ treatment, the SRWC of the W_+15_, W_0_ and W_−15_ treatments decreased to 63.6%, 59.7% and 57.8%, respectively. Simultaneously, the LWP assessed the degree of the water status and the changes in the SRWC. From Fig. 1, in the T_0_ treatment, the LWP of the W_+15_, W_0_ and W_−15_ treatments in S. *baicalensis* was −1.6, −1.8 and −1.9 MPa, respectively. At the temperature of T_4_, the LWP of the W_+15_, W_0_ and W_−15_ treatments decreased to −3.5, −3.7 and −3.8 MPa, respectively.

### Gas exchange parameters

**Figure 2:**
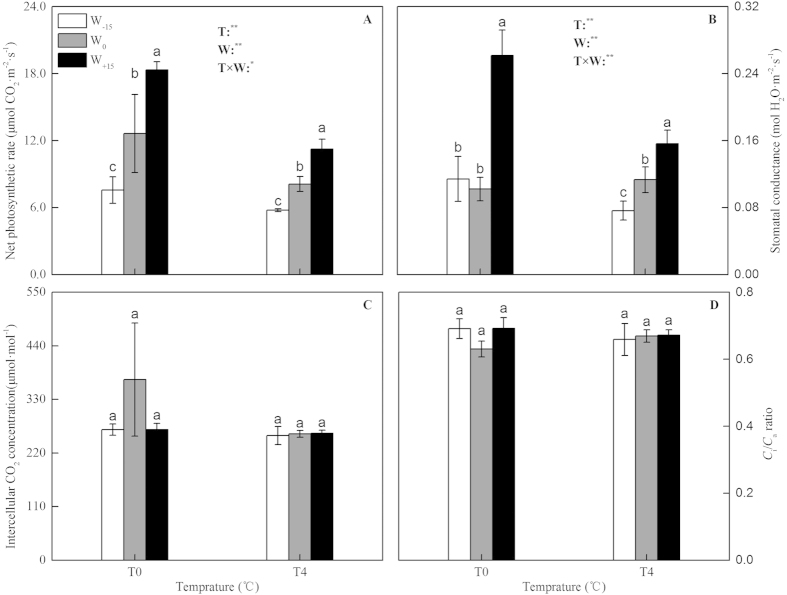
Combined effects of temperature and water availability on net photosynthetic rate (**A**), stomatal conductance (**B**), intercellular CO_2_ concentration(**C**) and *C*_i_/*C*_a_ ratio (**D**) of *Stipa baicalensis*. T, Temperature treatment; W, Precipitation treatment. Vertical bars represent ± SD of the mean (n = 3); Different letters on the SD bars indicate significant differences among the treatments (*p* < 0.05). ns, No significant differences. *Indicates significant differences at *p* < 0.05. **Indicates significant differences at *p* < 0.01.

From Fig. 2, the net CO_2_ assimilation rate (*P*_n_, Fig. 2A) and the stomatal conductance (*G*_s_, Fig. 2B) of S. *baicalensis* were significantly affected by both the temperature and water availability treatments (*p* < 0.05), whereas the effects of the treatments on the intercellular CO_2_ concentrations (*C*_i_, Fig. 2C) and the ratio of intercellular to ambient CO_2_ concentrations (the *C*_i_/*C*_a_ ratio, Fig. 2D) were not significant (*p* > 0.05). In the T_0_ treatment, compared with the W_0_ treatment, the *P*_n_ decreased significantly by 40.0% in the W_−15_ treatment, and in the W_+15_ treatment, the *P*_n_ increased by a large 45.3%. With the increase in temperature in the T_4_ treatment, compared with the W_0_ treatment, the *P*_n_ decreased by 28.8% in the W_−15_ treatment, and in the W_+15_ treatment, the *P*_n_ increased by 38.8%. However, under the different water treatments, compared with the T_0_ treatment, in the T_4_ treatment, the *P*_n_ significantly decreased by approximately 28.3–38.7%. In the T_0_ treatment, compared with the W_0_ treatment, the W_−15_ treatment had no significant effect on the *G*_s_, but in the W_+15_ treatment, the *G*_s_ increased by 1.5-fold. In the T_4_ treatment, compared with the W_0_ treatment, the *G*_s_ decreased by 32.5% in the W_−15_ treatment but the *G*_s_ increased by 1.5-fold in the W_+15_ treatment. Under the W_−15_, W_0_ and W_+15_ water availability treatments, compared with the T_0_ treatment, in the T_4_ treatment, the *G*_s_ decreased by 33.1%, increased by 10.8% and decreased by 40.3%, respectively.

### Leaf photosynthetic parameters

**Figure 3:**
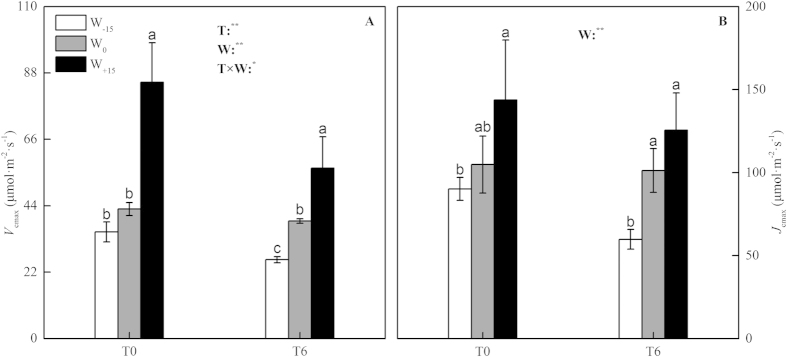
Combined effects of temperature and water availability on the maximum velocity of Rubisco carboxylation (**A**) and the maximum velocity of RuBP regeneration (**B**) in leaves of *Stipa baicalensis*. T, Temperature treatment; W, Precipitation treatment. Vertical bars represent ± SD of the mean (n = 3); Different letters on the SD bars indicate significant differences among the treatments (*p* < 0.05). Significance levels are reported in the figures as a significant tendency with *when *p* < 0.05 and with **when *p* < 0.01.

The values for the maximum rate of Rubisco (*V*_cmax_) and the maximum rate of ribulose-bisphosphate (RuBP) regeneration (*J*_max_) in the different treatments of temperature and water availability are shown in Fig. 3. From Fig. 3A, the temperature and the change in water availability significantly affected the *V*_cmax_, and a significant interaction between the two factors was detected (*p* < 0.05). In the T_0_ treatment, compared with the W_0_ treatment, the W_−15_ treatment had a significant effect on the *V*_cmax_, whereas in W_+15_ treatment, the increase in the *V*_cmax_ was approximately double. In the T_4_ treatment, the *V*_cmax_ was significantly affected by the availability of water. Compared with the W_0_ treatment, the *V*_cmax_ decreased by 32.9% in the W_−15_ treatment, and in the W_+15_ treatment, the *V*_cmax_ increased by 45.0%. The *J*_max_ was significantly affected by water availability (*p* < 0.01, Fig. 3B) but was not affected by high temperature. In theT_0_ treatment, compared with the W_0_ treatment, although the *J*_max_ decreased in the W_−15_ treatment and increased in the W_+15_ treatment, the difference between the treatments was not large. However, in the T_4_ treatment, compared with the W_0_ treatment, the decrease in the *J*_max_ was significant at 41.0% in the W_−15_ treatment, and although the *J*_max_ increased in the W_+15_ treatment, the difference was not significant.

### Light response curves

**Figure 4:**
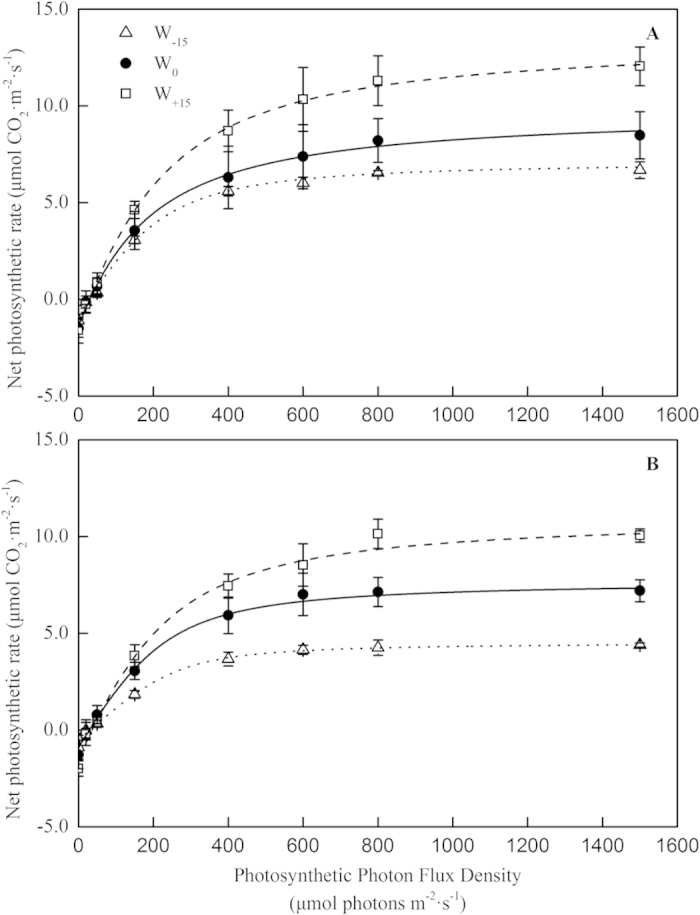
Photosynthetic light-response curves of leaves with a water deficit (open triangle symbol), the control leaves (closed circle symbol) and the leaves plus precipitation (open square symbol) of *Stipa baicalensis* growing at ambient temperature (**A**) and at high temperature (**B**).

The photosynthetic light response curves are a reflection of the ability of the plant to use light. The light response curves of S. *baicalensis* in the different treatments of temperature and water availability are illustrated in Fig. 4A,B. Within the range of natural light intensity, the *P*_n_ of the leaves increased with the increase in the PAR. When the level of PAR reached the light saturation point (LSP), the curve became stable. When the PAR exceeded 1500 μmol·m^−2^·s^−1^, the photosynthesis of S. *baicalensis* suffered from light-inhibition, and the curve trended downwards. As shown in the curves, the response of the *P*_n_ to the light intensity was different under the different temperature and water treatments. Based on the simulated analysis of the light response curves (Table 1), the light-saturated photosynthesis (*P*_max_) and the apparent quantum yield (AQY) in the leaves of S. *baicalensis* were significantly affected by the temperature and the change in water availability (*p* < 0.01), and the AQY was significantly affected by the interaction of the two environmental factors (*p* < 0.01). However, no significant effects on the light compensation point (LCP), the light saturated point (LSP) or the dark respiration (*R*_d_) (*p* > 0.05) were observed. In the T_0_ treatment, compared with the W_0_ treatment, the *P*_max_ and the AQY decreased by 26.4% and 18.8% in the W_−15_ treatment, respectively, whereas the *P*_max_ and the AQY increased by 37.3% and 21.9% in the W_+15_ treatment, respectively. In the T_4_ treatment, compared with the W_0_ treatment, the *P*_max_ and the AQY decreased by 38.0% and 37.0% in the W_−15_ treatment, respectively, whereas the *P*_max_ and the AQY increased by 44.6% and 33.3% in the W_+15_ treatments, respectively. Moreover, in the W_−15_, W_0_ and W_+15_ treatments, compared with the T_0_ treatment, the *P*_max_ decreased by 29.6%, 16.4% and 11.9% and the AQY decreased by 10.8%, 15.6% and 7.7%, respectively, in the T_4_ treatment.

## Discussion

The LWP has been widely used as an index for the water status of plants, and the LWP values reflect the ability of a plant to avoid dehydration^35^. In the present study, the LWP had a significant relationship with the SRWC (Fig. 1), which indicated that the availability of water had a direct effect on water status of the plant. After relative long-term water treatments (42 days), the SRWC decreased from approximately 75.4–78.4% to approximately 60.4–67.5%. Although an increase in water availability of 15% increased the SRWC by 6.2%, the S. *baicalensis* continued to suffer from a water deficit. Simultaneously, the temperature increase of 4 °C largely decreased both the SRWC and the LWP (Fig. 1), which indicated that high temperature exacerbated the adverse effects of the water deficit in S. *baicalensis* by increasing soil moisture evaporation and plant leaf evapotranspiration^36^.

The analysis of leaf gas exchange is an important method to detect the biochemical and stomatal mechanisms of leaves in response to environmental changes. In this study, the change of gas exchange parameters revealed that, although the *P*_n_ and *G*_s_ were significantly affected by both the temperature and water availability treatments (*p* < 0.01), no corresponding differences in the *C*_i_ were detected (*p* > 0.05). Thus, the S. *baicalensis* grown at different temperatures and with different levels of water availability had the same intercellular CO_2_ concentration for photosynthesis^37^. Furthermore, the *C*_i_/*C*_a_ ratio is a useful and effective index to evaluate the stomatal acclimation, and a change in the index directly reflects any changes in the relationship between the capacity for CO_2_ fixation and the *G*_s_^38^,^39^. If the stomata acclimated to changes in the environment independently, then the *C*_i_/*C*_a_ would change. In our study, the *C*_i_/*C*_a_ did not change significantly among the temperature and water treatments (*p* > 0.05). Because the values of *C*_i_ and *C*_i_/*C*_a_ were unaffected by the different temperature and moisture treatments, the nonstomatal limitation was likely the primary mechanism for the regulation of photosynthesis in S. *baicalensis*, which was a conclusion that was similar to that of a study on transgenic tobacco^40^.

The nonstomatal limitation of photosynthesis was reflected in the changes in photosynthetic capacity. In C3-plants, according to the photosynthesis model of Farquhar *el al*.^3^, the photosynthetic capacity is defined by two parameters: the maximum rate of carboxylation (*V*_cmax_) and the maximum rate of electron transport (*J*_max_). In our study, based on the analysis of variance (Fig. 3), the *V*_cmax_ and the *J*_max_ were significantly affected by the change in water availability (*p* < 0.01). A decrease in the water availability of 15% caused a decline in the photosynthetic capacity (*V*_cmax_ and *J*_max_) of S. *baicalensis*. These results were similar to the responses of plants to a water deficit that were found by other researchers^41^. The decrease in the *V*_cmax_ might result from the reduced amount of active Rubisco in soils under drought conditions^12^, and the reduction in the *J*_max_ might be related to the limited regeneration of RuBP caused by an insufficient supply of NADPH or ATP or the limited enzymatic activity of sedoheptulose-1,7-bisphosphatase and fructose-1,6-bisphosphatase under water stress conditions^10^,^17^. With an increase in water availability of 15%, the *V*_cmax_ and the *J*_max_ increased, and therefore, the increase in irrigation offset the negative effect of the drought, and the photosynthetic capacity of the S. *baicalensis* recovered, partially.

High temperatures inhibit the growth of plants and increase the respiration, which requires the plants to fix more carbon to sustain life. Based on previous reports, a temperature above 35 °C drastically limits photosynthesis by reducing the activity of Rubisco^42^. However, when plants are exposed to a long-term change in temperature, the acclimation of the photosynthetic apparatus possible occurs. By changing the optimum temperature for photosynthesis, photosynthesis can remain highly efficient at the new growth temperature^27^. Niu *et al*.^43^, for the species in a temperate steppe of northern China, found that elevated temperature increased the *V*_cmax_ and the *J*_max_ in the C3-grasses and the carboxylation efficiency (CE) and the CO_2_-saturated photosynthetic rate (*A*_sat_) in the C4-grass, which indicated that photosynthesis had acclimated to the elevated temperature. However, in our study, the effect of elevated temperature on the photosynthetic capacity was primarily caused by changes in the *V*_cmax_ (Fig. 4), which indicated that the effect of elevated temperature on the photosynthetic capacity of S. *baicalensis* was primarily caused by the reduction in the activation of Rubisco. This result was different from those that were found by other researchers, which could be explained by different plant species have different abilities to acclimate to a changing temperature. For cool-climate species (e.g., turnips, lamb’s-quarters, barley and broadbean), Bunce^44^ found that the *V*_cmax_ and the *J*_max_ were much higher for leaves grown at a cooler temperature than for those grown at a warmer temperature. The S. *baicalensis* grows in the high latitude regions of northern China and is highly acclimated to cooler temperatures. When the temperature was increased, the high temperature reduced *V*_cmax_ and resulted in the photosynthetic downregulation of S. *baicalensis*.

The light response curve of plant photosynthesis, which is important to understand the photochemical efficiency, has been widely used in research on plant physiology^45^. The parameters of the light response curve reflect the response mechanisms of plant photosynthesis to environmental factors such as drought^46^, elevated CO_2_ concentration^47^ and temperature^48^. The maximum photosynthetic rate (*P*_max_) is the maximum absolute value of photosynthesis under optimal environmental conditions^3^,^49^. The apparent quantum efficiency (AQY) is an indicator of the ability of a plant to absorb, covert and use light energy at low light intensities, and a high AQY indicates that plants have a high efficiency of light energy transfer^50^. One study showed that both a water deficit and flooding decreased the AQY^51^. In the present study, with the water availability decreased by 15% and the temperature increased by 4 °C, the *P*_max_ and AQY decreased, whereas an increase in water availability of 15% increased the values. This decrease in light use efficiency of S. *baicalensis* could be a mechanism for self-photoprotection that was triggered because of the contrasting requirements to dissipate heat and use energy for photochemical reactions^52^. With light saturation, the environmental stress suppressed the photochemical reactions within the chloroplasts, which resulted in excitation energy that could not be used in photochemical reactions. To protect the photosynthetic apparatus from damage by the excess excitation energy, the plant dissipates the excess excitation energy as harmless heat through the xanthophyll cycle^53^. With more heat dissipated, the light use was lower and the reduction in the photosynthetic rate was larger.

The LSP and the LCP are the two primary indicators of the demand of a plant for sunlight and are indicators of the ability of the plants to use high and low light intensities. When the environment is not suitable, plants typically reduce the LSP or improve the LCP to ensure the normal operation of photosynthesis. The *R*_d_ provides the energy for the activities of a plant^54^ and is the index of the respiration rate of plants in the dark^55^. Crous^56^ found that soil drought caused a decrease in the *R*_d_, and the author suggested that the possible reason was that the closing of the stomata under a water deficit changed the ratio of CO_2_ and O_2_ in the leaves and thereby affected the respiration of the plant^57^. However, the results from other studies also suggested that drought may inhibit the physiological activity of the plant, which reduces the requirement for energy and intermediate metabolites, resulting in the decrease of the *R*_d_^58^. Furthermore, short-term temperature elevations can increase the *R*_d_^59^ because a higher temperature could possibly increase the substrate concentration for the *R*_d_, which would lead to an increase in the activity of respiratory enzymes^60^. In our study, the relative long-term elevation in temperature and change in water availability had no significant effect on the *R*_d_, the LCP or the LSP of S. *baicalensis*, which indicated that the ability to use high and low light intensities for S. *baicalensis* under a changed environment had not yet responded, and the S. *baicalensis* remained capable of maintaining a normal metabolism with the dark respiration providing the energy for the plants.

## Conclusion

In conclusion, a decrease in the available soil water led to a water deficit in S. *baicalensis* after 42 days of irrigation. A temperature increase of 4 °C exacerbated the negative effects of the water deficit on S. *baicalensis*. Based on the measurements of the parameters of gas exchange, the nonstomatal limitation was the primary cause for the regulation of photosynthesis in S. *baicalensis*. The mechanism by which the increase in temperature and change in water availability affected photosynthesis in S. *baicalensis* was through the regulation of the photosynthetic capacity. With a decrease in water availability of 15%, the photosynthetic capacity decreased because of a reduction in the activation of Rubisco and the limitation of RuBP regeneration, which resulted in the nonstomatal limitation on S. *baicalensis*. An increase in water availability of 15% offset the negative effect of the drought and the photosynthetic capacity partially recovered. The mechanism by which a temperature higher by 4 °C affected the photosynthetic capacity of *S. baicalensis* was primarily through a change in the *V*_cmax_. Although the photosynthesis of S. *baicalensis* was inhibited because of an elevated temperature and a water deficit, the S. *baicalensis* maintained a normal metabolism with dark respiration providing the energy for the plants. Under environmental stress, the S. *baicalensis* had an apparent self-photoprotection mechanism to maintain its normal live activities.

## Methods

### Plant culture and experimental design

The experiment was conducted at the Institute of Botany, Chinese Academy of Sciences in 2012. The seeds of S. *baicalensis* were obtained from the grassland in Hailaer (49°22′N, 119°73′E), Inner Mongolia. Before sowing, the seeds were sterilized by soaking in a 0.7% potassium permanganate solution for 8 min; the seeds were rinsed following the sterilization. The plastic pots (10.9 cm in diameter, 9.5 cm in height), each wrapped with plastic film, were filled with approximately 0.61 kg of dry soil (organic carbon content 12.3 g·kg^−1^, total nitrogen content 1.45 g·kg^−1^ and soil field capacity 25.8%), and ten seeds were planted per pot. The pots were placed in a naturally illuminated glasshouse (the daytime/nighttime temperature was maintained at approximately 26–28 °C/18–20 °C, with a photosynthetic photon flux density of 1000 μmol·m^−2^·s^−1^ above the plant canopy) and were well watered to complete the growth of the seedlings. With the emergence of the third leaf (approximately 2 to 3 weeks after sowing), the seedlings were thinned to four plants per pot. Six replicates were used for the three water and the two temperature treatments. Then, 36 pots with healthy plants (four plants per pot) were randomly selected and placed into two environmental control chambers in which the two temperature treatments were simulated (Ambient temperature, T_0_; Ambient temperature +4 °C, T_4_) and the three water irrigation treatments were applied (Control, W_0_; W_0_ increased by 15%, W_+15_; W_0_ decreased by 15%, W_−15_). To avoid the effects from other environmental factors, such as light and relative humidity, the arrangements of the pots with different treatments were randomized once a week.

### Measurements

#### Soil relative water content

The soil relative water content (SRWC) (the ratio between the current soil moisture and the field capacity) was measured in the 0 to 8 cm soil layer. The measurement was determined 42 days after the plants were subjected to a relative long-term soil water treatment. Six replicates were used for each SRWC determination. The SRWC is expressed as follows:


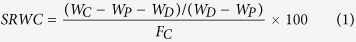


where *W*_*C*_ is the current soil weight, *W*_*P*_ is the empty pot weight (approximately 29.3 g), *W*_*D*_ is the dry soil weight, and *F*_*C*_ is the soil field capacity. *F*_*C*_ was measured after 24 hr after the soil was applied excessive water with six replicates according to method of Veihmeye & Hendrickson^61^.

### Leaf water potential

The leaf water potential of S. *baicalensis* was measured using a WP4 Dew-point Potential Meter (Decagon Device, Pullman, Washington). The leaf sample was obtained from a fully expanded leaf from the tip, with three plant replicates for each treatment. The measurements were conducted between 8:00 and 11:00 a.m. After cutting the sample, the leaf was immediately moved into the chamber and was equilibrated at least 10 min before determination of the leaf water potential.

### Leaf gas exchanges

Three plants from each treatment were selected from different pots, and the parameters of gas exchange were measured on the healthy and fully expanded leaves. The net photosynthetic rate (*P*_n_, μmol·CO_2_·m^−1^·s^−1^), stomatal conductance (*G*_s_, mol·m^−2^·s^−1^), intercellular CO_2_ concentration (*C*_i_, μmol·mol^−1^) and the ratio of intercellular to ambient CO_2_ concentration (the *C*_i_/*C*_a_ ratio) were measured using an open gas exchange system (LI-6400; Li-COR Inc., Lincoln, NE, USA) at the blooming stage of *S. breviflora* between 8:00~11:00 A.M. and 3:00~5:00 P.M. on sunny days. Because the leaves of *S. breviflora* were too narrow to cover an entire cuvette of the LI-6400, the area of the leaf was determined to recalculate the values for the parameters of gas exchange. The illumination was supplied to the leaves from a red-blue LED light source. The leaf chamber temperature was maintained at room temperature with a CO_2_ concentration of 390 ppm and a photosynthetic photon flux density (PPFD) of 900 μmol·m^−2^·s^−1^.

### Light response curves

The leaves used to obtain the light response curves were the identical leaves that were used for the determination of the parameters of gas exchange (see Supplementary Information: Photosynthetic irradiance response curves). The open gas exchange system (LI-6400) was also used to control the light intensity inside the leaf chamber. After a period of acclimation, the light curves were determined using the ‘Auto Light Curve Program’ with nine light intensities: 1500, 900, 600, 400, 150, 100, 50, 20, and 0 μmol·m^−2^·s^−1^ of photon irradiance. For the different light intensities, the wait time was at least 1 min. The light response curve was modelled by fitting the data to a nonrectangular hyperbola function^62^:


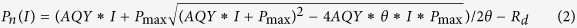


where *P*_n_ is the net photosynthetic rate (μmol·CO_2_·m^−1^·s^−1^), *P*_max_ is the light-saturated rate of CO_2_ accumulation (μmol·CO_2_·m^−1^·s^−1^), *I* is the photosynthetic photon flux density (μmol·m^−2^·s^−1^), AQY is the leaf maximum apparent quantum yield of CO_2_ uptake, *θ* is the convexity of the light response curve and *R*_d_ is the dark respiration (μmol·m^−2^·s^−1^). The quantum yield was estimated from the initial slope by applying linear regression to the low-photon flux data (less than 150 μmol·m^−2^s^−1^) of the light response curve. The intersection of the straight line with the X-axis corresponded to the light compensation point (LCP, μmol·m^−2^·s^−1^). The projection of *P*_max_ to the X-axis corresponded to the light saturated point (LSP, μmol·m^−2^·s^−1^).

### *A*/*C*_i_ curves

The measurements for the *A*/*C*_i_ curve were performed on the identical leaves that were used in the measurements of the parameters of gas exchange (see Supplementary Information: *A/C*_i_ curves). The *A*/*C*_i_ curve was generated under a light saturation level of 900 μmol·m^−2^·s^−1^ PPFD, and the leaf chamber was initially set to a CO_2_ concentration of 390 μmol·mol^−1^ for 10 min to ensure the activation of the Rubisco under steady-state conditions. The CO_2_ gradients for *A*/*C*_i_ curves included the following stepwise levels: 390, 200, 100, 50, 390, 600, 800, and 1000 μmol·mol^−1^. The curve-fitting software by Sharkey *et al*.^63^ was used in the analysis of the *A*/*C*_i_ curve, which produced a revised model based on the version of Farquhar^3^. A nonlinear regression technique was used to estimate the *V*_cmax_ and the *J*_max_, and the values were standardized to the leaf temperature of 25 °C, as suggested by Sharkey *et al*.^63^.

### Statistical analyses

The parameters described above were measured at the blooming stage (42 days after the plants were subjected to the temperature and soil water treatments of S. *breviflora*. The statistical analyses were performed using the SPSS 18.0 statistical software package (SPSS, Chicago, IL, USA). For each treatment, the mean with standard deviation (±SD) was shown. The effects of temperature or water availability on the photosynthetic parameters were determined with one-way analysis of variance (ANOVA, *p* < 0.05), followed by Duncan’s multiple range tests (Duncan, 1955). The significance of the interaction effect of temperature and water availability on the photosynthetic parameters was analysed using a two-way ANOVA (*p* < 0.05). The graphs were constructed using the Origin 9.0 software (Origin Lab, USA).

## Additional Information

**How to cite this article**: Song, X. *et al*. A self-photoprotection mechanism helps *Stipa baicalensis* adapt to future climate change. *Sci. Rep.* **6**, 25839; doi: 10.1038/srep25839 (2016).

## Supplementary Material

Supplementary Information

## Figures and Tables

**Table 1:** Combined effects of warming and water availability on light-saturated photosynthesis, apparent quantum yield, light compensation point, light saturated point and dark respiration in leaves of *Stipa baicalensis*.

Treatment	T_0_			T_4_			*p*		
	W_−15_	W_0_	W_+15_	W_−15_	W_0_	W_+15_	T	W	T×W
*P*_max_ (μmol·m^−2^·s^−1^)	8.1 ± 0.3^c^	11.0 ± 0.5^b^	15.1 ± 1.2^a^	5.7 ± 0.4^c^	9.2 ± 0.4^b^	13.3 ± 0.3^a^	**	**	ns
AQY (mol·mol^−1^)	0.026 ± 0.003^b^	0.032 ± 0.005^ab^	0.039 ± 0.008^a^	0.017 ± 0.005^c^	0.027 ± 0.006^b^	0.036 ± 0.004^a^	**	**	**
LCP (μmol·m^−2^·s^−1^)	35.5 ± 4.3^a^	36.4 ± 15.9^a^	31.4 ± 11.0^a^	33.1 ± 18.3^a^	31.4 ± 19.2^a^	41.8 ± 7.9^a^	ns	ns	ns
LSP (μmol·m^−2^·s^−1^)	349.5 ± 33.0^a^	386.7 ± 52.4^a^	423.2 ± 55.1^a^	383.9 ± 46^a^	380.7 ± 48.7^a^	418.6 ± 41.4^a^	ns	ns	ns
*R*_d_ (μmol·m^−2^·s^−1^)	1.0 ± 0.2^a^	1.4 ± 0.5^a^	1.1 ± 0.2^a^	0.7 ± 0.6^a^	1.0 ± 0.6^a^	1.7 ± 0.2^a^	ns	ns	ns

ns, No significant differences.

^*^Indicates significant differences at *p* < 0.05.

^**^Indicates significant differences at *p* < 0.01.
