# Supplementary material for: A self-photoprotection mechanism helps Stipa baicalensis adapt to future climate change
Source: Sci Rep. 2016 May 10;6:25839. doi: 10.1038/srep25839 (PMC4861908; doi:10.1038/srep25839)
Supplement: Supplementary Information [file srep25839-s1.pdf]

A self-photoprotection mechanism helps *Stipa baicalensis* adapt to future climate change

Xiliang Song[1,3], Guangsheng Zhou[2,1]*, Zhenzhu Xu[1,3], Xiaomin Lv[1,3], Yuhui Wang[1,3*]

[1]State Key Laboratory of Vegetation and Environmental Change, Institute of Botany, Chinese Academy of Science, 20 Nanxincun, Xiangshan, Beijing 100093, China; [2]Chinese Academy of Meteorological Sciences, China Meteorological Administration, 46 Zhongguancun South Street, Haidian, Beijing 100081, China; [3]University of Chinese Academy of Sciences, 19A Yuquan Road, Beijing 100049, China.

*Author for correspondence: Guangsheng Zhou, Tel.: 86(10)13621097075, Fax: 86(10)82595962, E-mail: gszhou@ibcas.ac.cn; Yuhui Wang, Tel.: 86(10)13522893410; Fax: 86(10)82595962; E-mail: yhwang@ibcas.ac.cn

Photosynthetic irradiance response curves

**T_0 ×W_{-15}**			
		PAR	Photo
		μmol·m^{-2} s^{-1}	μmol·m^{-2} s^{-1}
***Stipa baicalensis*-1**			
1		1500	6.2
2		900	6.6
3		600	6.0
4		400	5.5
5		150	3.3
6		50	0.6
7		20	0.0
8		0	-1.3
***Stipa baicalensis*-2**			
9		1500	6.7
10		900	6.4
11		600	5.7
12		400	5.4
13		150	2.9
14		50	0.3
15		20	-0.2
		0	-0.7
***Stipa baicalensis*-3**			
16		1500	7.1
17		900	6.6
18		600	6.3
19		400	5.9
20		150	3.0
21		50	0.1
22		20	-0.2
23		0	-1.3

**$T_0 \times W_0$**			
		PAR	Photo
		μmol·m^{-2} s^{-1}	μmol·m^{-2} s^{-1}
***Stipa baicalensis*-1**			
1		1500	7.8
2		900	7.6
3		600	6.4
4		400	5.7
5		150	3.0
6		50	0.7
7		20	-0.1
8		0	-1.4
***Stipa baicalensis*-2**			
9		1500	7.8
10		900	7.5
11		600	6.5
12		400	5.1
13		150	3.0
14		50	0.2
15		20	-0.7
16		0	-2.0
***Stipa baicalensis*-3**			
17		1500	9.9
18		900	9.5
19		600	9.3
20		400	8.1
21		150	4.7
22		50	1.1
23		20	0.4
24		0	-1.1

$T_0 \times W_{+15}$			
		PAR	Photo
		μmol·m^{-2} s^{-1}	μmol·m^{-2} s^{-1}
***Stipa baicalensis*-1**			
1		1500	11.3
2		900	10.9
3		600	9.6
4		400	8.5
5		150	4.6
6		50	0.9
7		20	-0.2
8		0	-1.5
***Stipa baicalensis*-2**			
9		1500	11.6
10		900	10.2
11		600	9.2
12		400	7.8
13		150	4.2
14		50	1.3
15		20	0.1
16		0	-1.0
***Stipa baicalensis*-3**			
17		1500	13.2
18		900	12.7
19		600	12.2
20		400	9.9
21		150	5.1
22		50	0.2
23		20	-0.7
24		0	-2.3

$T_4 \times W_{-15}$			
		PAR	Photo
		μmol·m^{-2} s^{-1}	μmol·m^{-2} s^{-1}
***Stipa baicalensis*-1**			
1		1500	4.5
2		900	4.4
3		600	4.2
4		400	3.6
5		150	1.9
6		50	0.3
7		20	0.3
8		0	-0.4
***Stipa baicalensis*-2**			
9		1500	4.3
10		900	3.8
11		600	3.9
12		400	3.4
13		150	1.6
14		50	0.5
15		20	0.1
16		0	-0.7
***Stipa baicalensis*-3**			
17		1500	4.5
18		900	4.6
19		600	4.3
20		400	4.1
21		150	2.0
22		50	0.2
23		20	-0.4
24		0	-1.6

$T_4 \times W_0$			
		PAR	Photo
		μmol·m^{-2} s^{-1}	μmol·m^{-2} s^{-1}
***Stipa baicalensis*-1**			
1		1500	6.8
2		900	6.3
3		600	5.9
4		400	4.9
5		150	2.6
6		50	0.7
7		20	0.0
8		0	-1.0
***Stipa baicalensis*-2**			
9		1500	6.9
10		900	7.4
11		600	8.1
12		400	6.7
13		150	3.2
14		50	0.4
15		20	-0.9
16		0	-2.1
***Stipa baicalensis*-3**			
17		1500	7.9
18		900	7.7
19		600	7.1
20		400	6.2
21		150	3.4
22		50	1.3
23		20	0.4
24		0	-0.7

**$T_4 \times W_{+15}$**			
		PAR	Photo
		μmol·m^{-2} s^{-1}	μmol·m^{-2} s^{-1}
***Stipa baicalensis*-1**			
1		1500	9.7
2		900	9.3
3		600	7.5
4		400	6.8
5		150	3.2
6		50	0.2
7		20	-0.4
8		0	-1.8
***Stipa baicalensis*-2**			
9		1500	10.3
10		900	10.6
11		600	9.7
12		400	8.0
13		150	4.0
14		50	0.3
15		20	-0.4
16		0	-2.5
***Stipa baicalensis*-3**			
17		1500	10.2
18		900	10.6
19		600	8.5
20		400	7.5
21		150	4.3
22		50	0.7
23		20	0.1
24		0	-1.7

A_Ci_curves

$T_0 \times W_{-15}$														
	Cond	Trmmol	Area	Tleaf	CO2R	CO2S	H2OR	H2OS	RH_R	RH_S	Flow	PARi	**Photo**	**Ci**
	mmol m^{-2} s^{-1}	mmol m^{-2} s^{-1}	cm^2	℃	μmol·mol^{-1}	μmol·mol^{-1}	mb	mb	%	%	ml min^{-1}	μmol·m^{-2} s^{-1}	**μmol·m^{-2} s^{-1}**	**μmol·mol^{-1}**
***Stipa baicalensis*-1**														
1	0.109	2.5	2	26.2	55	55	10.6	11.2	30.2	32.0	301	902	**-1.0**	**68.3**
2	0.095	2.2	2	26.2	99	99	10.6	11.1	30.1	31.7	301	902	**0.3**	**90.7**
3	0.079	1.8	2	26.2	200	199	10.5	11.0	30.0	31.4	301	903	**2.2**	**148.5**
4	0.117	2.7	2	26.3	391	387	10.6	11.3	30.2	32.2	301	902	**10.7**	**228.2**
5	0.102	2.4	2	26.4	600	595	10.7	11.3	30.3	32.0	301	902	**15.3**	**337.5**
6	0.087	2.0	2	26.5	799	794	10.7	11.2	30.2	31.8	301	902	**18.6**	**427.2**
7	0.075	1.8	2	26.6	1001	995	10.7	11.1	30.2	31.5	300	901	**20.7**	**523.3**
***Stipa baicalensis*-2**														
8	0.054	0.9	2	25.8	56	56	17.3	17.6	62.6	63.7	300	898	**-1.1**	**88.0**
9	0.045	0.7	2	25.8	100	100	17.1	17.4	62.1	63.1	300	899	**-0.5**	**116.3**
10	0.037	0.6	2	25.7	200	199	16.9	17.1	61.6	62.4	300	899	**0.6**	**166.5**
11	0.066	1.0	2	25.9	390	388	17.5	17.9	63.4	64.8	300	898	**5.2**	**254.0**
12	0.067	1.0	2	25.9	600	597	17.7	18.0	63.8	65.1	300	898	**8.8**	**373.8**
13	0.066	1.0	2	26.0	800	795	17.7	18.1	63.8	65.2	300	897	**11.2**	**507.5**
14	0.056	0.9	2	26.1	1001	996	17.8	18.1	64.0	65.2	300	897	**12.0**	**635.1**
***Stipa baicalensis*-3**														
15	0.117	2.6	2	26.1	55	55	10.7	11.7	30.4	33.1	301	899	**-0.4**	**58.9**
16	0.105	2.4	2	26.2	99	99	10.7	11.6	30.4	32.8	300	899	**0.6**	**85.9**
17	0.088	2.0	2	26.3	200	199	10.7	11.4	30.3	32.3	300	899	**3.2**	**134.0**
18	0.125	2.7	2	26.1	390	386	10.7	11.7	30.4	33.3	301	899	**9.9**	**246.6**

19	0.119	2.6	2	26.0	600	594	10.7	11.7	30.4	33.2	301	900	**14.3**	**382.8**
20	0.104	2.3	2	26.0	799	793	10.7	11.5	30.4	32.8	300	900	**16.3**	**516.3**
21	0.084	1.9	2	26.1	1001	994	10.7	11.4	30.4	32.4	300	900	**17.9**	**623.9**

T_0×W_0														
	Cond	Trmmol	Area	Tleaf	CO2R	CO2S	H2OR	H2OS	RH_R	RH_S	Flow	PARi	**Photo**	**Ci**
	mmol m^{-2} s^{-1}	mmol m^{-2} s^{-1}	cm^2	℃	μmol·mol^{-1}	μmol·mol^{-1}	mb	mb	%	%	ml min^{-1}	μmol·m^{-2} s^{-1}	**μmol·m^{-2} s^{-1}**	**μmol·mol^{-1}**
***Stipa baicalensis*-1**														
1	0.223	4.7	2	26.3	55	55	12.3	13.0	37.6	39.9	300	900	**-2.5**	**70.8**
2	0.204	4.3	2	26.3	99	99	12.2	12.9	37.4	39.5	301	900	**0.1**	**94.7**
3	0.179	3.9	2	26.3	200	199	12.1	12.7	37.2	39.1	301	901	**4.0**	**157.3**
4	0.206	4.4	2	26.4	390	387	12.4	13.1	38.0	40.1	301	900	**15.6**	**254.2**
5	0.195	4.2	2	26.4	600	596	12.5	13.1	38.2	40.2	300	900	**23.5**	**385.3**
6	0.177	3.8	2	26.6	799	794	12.6	13.2	38.4	40.2	300	900	**28.5**	**512.1**
7	0.164	3.6	2	26.6	1001	995	12.6	13.2	38.6	40.3	300	900	**32.0**	**653.6**
***Stipa baicalensis*-2**														
8	0.138	2.2	2	24.8	55	55	14.9	15.7	50.2	52.9	300	901	**-0.9**	**63.6**
9	0.126	2.0	2	24.7	100	100	14.8	15.5	49.9	52.4	300	902	**0.6**	**89.4**
10	0.119	1.9	2	24.6	200	198	14.7	15.3	49.7	52.0	300	902	**3.3**	**148.8**
11	0.155	2.4	2	24.9	390	386	15.1	16.0	50.8	53.8	301	900	**9.2**	**281.8**
12	0.153	2.4	2	25.0	600	594	15.3	16.2	51.2	54.1	301	900	**13.7**	**437.5**
13	0.147	2.3	2	25.0	800	793	15.4	16.2	51.5	54.3	301	900	**16.9**	**589.7**
14	0.137	2.1	2	25.0	1001	993	15.5	16.2	51.9	54.5	300	900	**18.5**	**753.6**
***Stipa baicalensis*-3**														
15	0.064	1.5	2	24.1	56	57	6.0	6.5	19.0	20.8	301	898	**-1.5**	**91.3**
16	0.059	1.4	2	24.0	101	101	5.8	6.4	18.6	20.3	301	899	**-0.8**	**117.6**
17	0.054	1.3	2	24.0	200	200	5.7	6.2	18.3	19.8	301	899	**0.9**	**164.9**
18	0.068	1.5	2	23.8	389	387	6.2	6.7	19.7	21.5	301	899	**5.2**	**251.3**
19	0.065	1.5	2	23.7	600	596	6.2	6.8	20.0	21.7	301	900	**9.0**	**357.1**

20	0.059	1.3	2	23.7	800	795	6.3	6.8	20.2	21.8	301	899	**10.8**	**478.1**
21	0.054	1.3	2	24.2	1001	995	6.4	6.8	20.3	21.8	301	897	**13.1**	**571.9**

**T_0 ×W+$_{15}$**														
	Cond	Trmmol	Area	Tleaf	CO2R	CO2S	H2OR	H2OS	RH_R	RH_S	Flow	PARi	**Photo**	**Ci**
	mmol m^{-2} s^{-1}	mmol m^{-2} s^{-1}	cm^2	℃	μmol·mol^{-1}	μmol·mol^{-1}	mb	mb	%	%	ml min^{-1}	μmol·m^{-2} s^{-1}	**μmol·m^{-2} s^{-1}**	**μmol·mol^{-1}**
***Stipa baicalensis*-1**														
1	0.145	3.4	2	24.8	56	56	6.8	7.7	21.3	24.1	301	903	**-1.0**	**64.8**
2	0.135	3.2	2	24.8	100	100	6.8	7.6	21.3	23.9	300	897	**0.1**	**94.5**
3	0.120	2.8	2	24.8	200	199	6.8	7.6	21.4	23.7	301	898	**3.5**	**145.5**
4	0.140	3.4	2	25.0	391	388	6.8	7.7	21.2	23.9	301	902	**9.4**	**267.0**
5	0.131	3.1	2	25.0	599	595	6.8	7.7	21.3	23.9	301	902	**13.3**	**412.4**
6	0.112	2.7	2	25.2	799	795	6.9	7.6	21.6	23.8	301	902	**15.5**	**545.9**
7	0.097	2.4	2	25.2	1001	996	6.9	7.5	21.5	23.4	301	902	**16.4**	**692.9**
***Stipa baicalensis*-2**														
8	0.298	5.5	2	23.5	55	55	9.2	10.4	29.5	33.1	301	900	**-0.5**	**56.0**
9	0.281	5.2	2	23.4	99	99	9.2	10.3	29.5	32.9	301	900	**2.5**	**81.6**
10	0.244	4.6	2	23.5	200	198	9.2	10.2	29.3	32.4	301	900	**8.4**	**137.3**
11	0.286	5.4	2	23.6	390	385	9.2	10.4	29.4	33.0	301	899	**21.4**	**254.3**
12	0.261	5.0	2	23.8	600	594	9.3	10.3	29.4	32.7	301	899	**28.6**	**401.9**
13	0.211	4.1	2	24.0	800	792	9.3	10.2	29.6	32.4	301	899	**32.3**	**525.4**
14	0.168	3.4	2	24.2	1001	993	9.4	10.1	29.8	32.1	301	898	**33.9**	**642.4**
***Stipa baicalensis*-3**														
15	0.090	2.4	2	26.2	57	58	7.2	8.0	21.9	24.2	300	898	**-0.9**	**70.6**
16	0.067	1.8	2	26.3	101	101	7.2	7.8	22.0	23.7	301	898	**-0.2**	**101.0**
17	0.043	1.2	2	26.5	200	199	7.3	7.6	22.0	23.1	301	898	**0.9**	**158.3**
18	0.061	1.6	2	26.4	390	388	7.2	7.7	21.9	23.5	301	898	**6.0**	**215.9**
19	0.051	1.4	2	26.4	600	597	7.2	7.6	21.9	23.2	300	898	**9.1**	**289.7**

20	0.039	1.1	2	26.4	799	796	7.2	7.5	21.9	22.9	301	898	**11.1**	**312.4**
21	0.033	0.9	2	26.4	1001	996	7.2	7.5	21.9	22.8	300	898	**13.2**	**335.8**

$T_4 \times W_{-15}$														
	Cond	Trmmol	Area	Tleaf	CO2R	CO2S	H2OR	H2OS	RH_R	RH_S	Flow	PARi	**Photo**	**Ci**
	mmol m^{-2} s^{-1}	mmol m^{-2} s^{-1}	cm^2	℃	μmol·mol^{-1}	μmol·mol^{-1}	mb	mb	%	%	ml min^{-1}	μmol·m^{-2} s^{-1}	**μmol·m^{-2} s^{-1}**	**μmol·mol^{-1}**
***Stipa baicalensis*-1**														
1	0.052	1.3	2	28.7	56	56	15.4	15.7	41.3	42.1	300	897	**-1.5**	**100.8**
2	0.052	1.2	2	28.6	101	101	15.4	15.7	41.3	42.2	300	898	**-0.8**	**121.0**
3	0.043	1.0	2	28.6	200	200	15.5	15.8	41.6	42.3	301	897	**0.6**	**170.7**
4	0.057	1.4	2	28.7	391	389	15.3	15.7	41.1	42.1	300	903	**5.5**	**221.3**
5	0.056	1.4	2	28.7	599	597	15.3	15.6	40.9	41.8	300	903	**7.4**	**366.7**
6	0.052	1.3	2	28.6	800	797	15.2	15.6	40.8	41.7	300	897	**9.6**	**477.5**
7	0.046	1.1	2	28.6	1001	998	15.2	15.5	40.8	41.6	300	903	**10.2**	**610.5**
***Stipa baicalensis*-2**														
8	0.114	2.4	2	27.8	56	56	15.5	16.3	42.2	44.3	301	898	**-1.2**	**70.6**
9	0.101	2.2	2	27.8	101	101	15.4	16.1	42.0	43.9	301	898	**-0.3**	**101.4**
10	0.086	1.9	2	27.9	200	199	15.4	16.0	41.8	43.4	301	898	**1.8**	**159.9**
11	0.126	2.7	2	27.8	390	387	15.5	16.4	42.2	44.5	301	898	**7.6**	**278.2**
12	0.124	2.7	2	28.0	599	595	15.6	16.5	42.3	44.6	300	903	**11.2**	**431.7**
13	0.109	2.4	2	28.1	799	795	15.6	16.4	42.3	44.3	300	902	**13.6**	**569.1**
14	0.094	2.1	2	28.1	1000	995	15.6	16.3	42.3	44.1	301	902	**15.0**	**708.9**
***Stipa baicalensis*-3**														
15	0.184	2.6	2	23.6	56	56	14.7	15.2	55.5	57.5	301	902	**-1.6**	**68.1**
16	0.178	2.5	2	23.6	100	100	14.7	15.2	55.4	57.4	301	902	**-0.2**	**99.0**
17	0.172	2.4	2	23.5	200	199	14.7	15.2	55.6	57.5	301	902	**2.8**	**169.0**
18	0.188	2.7	2	23.7	390	388	14.6	15.1	55.0	57.1	301	901	**9.0**	**303.3**
19	0.184	2.6	2	23.7	600	597	14.5	15.1	54.7	56.8	301	901	**14.1**	**460.6**

20	0.176	2.5	2	23.8	800	796	14.5	15.1	54.7	56.7	301	901	**17.6**	**617.9**
21	0.169	2.5	2	23.9	1001	996	14.5	15.0	54.4	56.3	301	900	**20.0**	**784.0**

**$T_4 \times W_0$**														
	Cond	Trmmol	Area	Tleaf	CO2R	CO2S	H2OR	H2OS	RH_R	RH_S	Flow	PARi	**Photo**	**Ci**
	mmol m^{-2} s^{-1}	mmol m^{-2} s^{-1}	cm^2	℃	μmol·mol^{-1}	μmol·mol^{-1}	mb	mb	%	%	ml min^{-1}	μmol·m^{-2} s^{-1}	**μmol·m^{-2} s^{-1}**	**μmol·mol^{-1}**
***Stipa baicalensis*-1**														
1	0.100	3.2	2	29.9	56	57	9.4	10.3	24.8	27.0	300	901	**-1.5**	**76.5**
2	0.090	2.9	2	29.9	101	101	9.6	10.3	25.2	27.2	301	901	**-0.3**	**100.6**
3	0.078	2.5	2	30.0	200	199	9.7	10.4	25.6	27.3	301	901	**1.7**	**156.0**
4	0.072	2.4	2	30.3	389	387	9.4	10.0	24.6	26.3	300	901	**6.7**	**223.4**
5	0.053	1.8	2	30.5	600	597	9.4	9.8	24.6	25.8	300	900	**8.9**	**305.7**
6	0.049	1.7	2	30.4	799	796	9.3	9.8	24.5	25.7	300	901	**12.2**	**366.6**
7	0.047	1.6	2	30.6	1000	996	9.3	9.8	24.4	25.6	300	900	**14.3**	**471.9**
***Stipa baicalensis*-2**														
8	0.061	1.1	2	23.7	55	55	11.4	11.6	40.7	41.3	301	902	**-2.2**	**109.2**
9	0.052	0.9	2	23.8	99	99	11.3	11.4	40.3	40.8	301	902	**-1.6**	**145.6**
10	0.065	1.1	2	23.7	390	388	11.6	11.8	41.4	42.1	301	903	**7.9**	**184.7**
11	0.061	1.1	2	23.7	600	598	11.7	11.9	41.8	42.4	301	903	**11.5**	**281.9**
12	0.052	0.9	2	23.6	800	797	11.8	11.9	42.1	42.6	300	903	**14.5**	**333.3**
13	0.045	0.8	2	23.7	1001	998	11.9	12.0	42.4	42.9	301	903	**17.3**	**367.4**
***Stipa baicalensis*-3**														
14	0.077	1.8	2	25.7	55	55	9.6	10.1	27.5	28.8	301	899	**-1.1**	**75.4**
15	0.069	1.6	2	25.8	100	100	9.6	10.0	27.3	28.5	301	899	**0.1**	**95.0**
16	0.062	1.5	2	25.8	200	199	9.5	9.9	27.2	28.3	300	899	**1.9**	**144.0**
17	0.088	2.0	2	25.7	390	387	9.7	10.2	27.7	29.2	300	899	**9.2**	**209.0**
18	0.089	2.0	2	25.7	599	595	9.8	10.3	27.9	29.4	301	899	**14.3**	**321.5**
19	0.082	1.9	2	25.8	799	794	9.8	10.3	27.9	29.4	300	899	**17.7**	**426.2**

**20**	0.071	1.6	2	25.8	1000	994	9.8	10.2	28.0	29.2	301	899	**19.6**	**525.4**

T_4×W_{+15}														
	Cond	Trmmol	Area	Tleaf	CO2R	CO2S	H2OR	H2OS	RH_R	RH_S	Flow	PARi	**Photo**	**Ci**
	mmol m^{-2} s^{-1}	mmol m^{-2} s^{-1}	cm^{2}	℃	μmol·mol^{-1}	μmol·mol^{-1}	mb	mb	%	%	ml min^{-1}	μmol·m^{-2} s^{-1}	**μmol·m^{-2} s^{-1}**	**μmol·mol^{-1}**
***Stipa baicalensis*-1**														
1	0.069	0.9	2	23.3	50	50	14.9	15.3	51.5	53.0	301	898	**-0.5**	**61.6**
2	0.061	0.8	2	23.3	100	100	15.0	15.3	51.7	53.0	301	898	**0.2**	**92.5**
3	0.053	0.7	2	23.3	200	199	15.1	15.4	52.1	53.3	301	898	**1.2**	**159.3**
4	0.046	0.6	2	23.3	389	388	15.2	15.5	52.6	53.6	301	898	**3.2**	**268.1**
5	0.081	1.1	2	23.1	600	596	14.6	15.1	50.7	52.4	301	899	**8.6**	**413.5**
6	0.076	1.0	2	23.0	800	794	14.5	15.0	50.4	52.1	301	899	**10.4**	**557.9**
7	0.066	0.9	2	23.1	1001	995	14.5	14.9	50.2	51.7	301	899	**11.7**	**690.7**
***Stipa baicalensis*-2**														
8	0.145	3.4	2	24.8	56	56	6.8	7.7	21.3	24.1	301	903	**-1.0**	**64.8**
9	0.135	3.2	2	24.8	100	100	6.8	7.6	21.3	23.9	300	897	**0.1**	**94.5**
10	0.120	2.8	2	24.8	200	199	6.8	7.6	21.4	23.7	301	898	**3.5**	**145.5**
11	0.153	3.6	2	24.9	390	388	6.8	7.8	21.2	24.2	301	902	**9.6**	**273.8**
12	0.131	3.1	2	25.0	599	595	6.8	7.7	21.3	23.9	301	902	**13.3**	**412.4**
13	0.112	2.7	2	25.2	799	795	6.9	7.6	21.6	23.8	301	902	**15.5**	**545.9**
14	0.097	2.4	2	25.2	1001	996	6.9	7.5	21.5	23.4	301	902	**16.4**	**692.9**
***Stipa baicalensis*-3**														
15	0.184	4.8	2	26.8	57	57	7.5	9.0	22.4	27.0	301	901	**-0.8**	**61.4**
16	0.174	4.6	2	26.9	101	101	7.6	9.0	22.5	26.9	301	902	**0.8**	**89.6**
17	0.158	4.2	2	26.9	200	198	7.6	8.9	22.6	26.6	301	902	**4.0**	**150.6**
18	0.178	4.7	2	26.7	389	385	7.5	9.0	22.3	26.8	300	902	**10.6**	**274.5**
19	0.124	3.4	2	27.0	600	595	7.4	8.5	22.2	25.4	301	902	**14.5**	**384.9**

20	0.106	3.0	2	27.1	799	793	7.4	8.3	22.1	24.9	301	902	**17.1**	**504.7**
21	0.100	2.8	2	27.1	1001	994	7.4	8.2	22.0	24.7	301	903	**18.5**	**660.4**
